# Panretinal acute multifocal placoid pigment epitheliopathy: a novel posterior uveitis syndrome with HLA-A3 and HLA-C7 association

**DOI:** 10.1186/1869-5760-3-29

**Published:** 2013-02-04

**Authors:** Kevin R Baxter, E Mitchel Opremcak

**Affiliations:** 1 Joseph H. Wyatt Ophthalmology Residency Program, St. John Providence Health System, 27351 Dequindre, Madison Heights, MI 48071, USA; 2 Patrick G. Murray Eye Center, 4777 E. Outer Drive, Detroit, MI 48234, USA; 3 The Retina Group, 262 Neil Ave, Ste 220, Columbus, OH, 43215, USA

**Keywords:** Acute posterior multifocal placoid pigment epitheliopathy, Uveitis, Retinal lesions

## Abstract

**Background:**

The purpose of this study is to report a previously undescribed clinical entity resembling acute posterior multifocal placoid pigment epitheliopathy (APMPPE) but with an atypical, panretinal and diffuse presentation in young patients with an HLA-A3 and HLA-C7 association. We describe a cluster of three, young, healthy patients who experienced acute visual loss, aged 16 through 27 years exhibiting an unusual clinical entity over an 8-month period.

**Findings:**

Our patients demonstrated a unique presentation with acute retinal lesions similar to APMPPE but had widespread presentation of multiple lesions in the peripheral retina. All three of our patients exhibited an acute loss of vision, two of them bilaterally. Ophthalmoscopy and fundus photography demonstrated a diffuse distribution of lesions located extensively throughout the retina. On fluorescein angiography, the lesions showed a characteristic ‘blocking early and staining late’ pattern similar to APMPPE. The average duration of activity was 6 weeks (range 4 to 8 weeks), and there were no recurrences and good visual prognosis. HLA-A3 and HLA-C7 was noted in 100% of the patients. Ocular coherence tomography during the acute stage in one patient demonstrated thickening at the RPE layer.

**Conclusions:**

To our knowledge, this cluster of young patients represents a previously undescribed clinical entity, with clinical features similar to APMPPE, relentless placoid chorioretinitis and ampiginous. Due to the diffuse distributions of the active lesions, the acute clinical course without recurrences, good visual prognosis, and HLA-A3/C7 association, we believe it to be distinct from these other white dot syndromes. All three patients experienced preceding viral-like prodrome which, when combined with major histocompatibility commonalities, may predispose these individuals to an immune response. We call this entity panretinal acute multifocal placoid pigment epitheliopathy or PAMPPE.

## Findings

### Background

*Acute posterior multifocal placoid pigment epitheliopathy* (APMPPE) was first described by Gass, is used to describe an inflammatory disease that affects the fundus of healthy young adults, both males and females, and is associated with an acute onset temporary visual loss [1-3]. APMPPE is characterized by the development of multiple gray-white, flat plaque-like lesions that are located in the posterior pole at the level of the retinal pigment epithelium (RPE) or choriocapillaris [1]. The plaques vary in size, but are clearly defined. Clinically, the disease presents with sudden onset of visual blurring and/or flashing lights in patients younger than 30 years of age [3]. It may be preceded with prodromal viral illness, along with headache and erythema nodosum [2,4]. Most cases are bilateral, but unilateral presentations with delayed appearance of disease in the second eye within several weeks have been noted. Typically, APMPPE is a nonrecurring disorder, with relatively good visual prognosis as 80% of patients regain 20/40 or better visual acuity. Recurrences are rare [3]. Associations have previously been reported with HLA-B7 and HLA-DR2 [5,6].

Other clinical entities that have been described in the literature are *relentless placoid chorioretinitis* (RPC) and *ampiginous chorioretinopathy*. These diseases have characteristics similar to APMPPE and serpiginous choroiditis but with atypical and chronic or recurrent clinical course [7]. The lesions can be unilateral or bilateral, with abrupt vision loss in the affected eye and delayed onset possibly occurring in the other eye. RPC has been reported to affect healthy men and women between the ages 16 and 51 years [7,8]. Previous publications report that there is typically greater than 50 lesions throughout the retina with RPC. Jones and co-investigators have reported that the retinal lesions seen with RPC resemble APMPPE and serpiginous choroiditis, but the lesions occurred in distinctive retinal distribution with prolonged relapsing clinical course. New active lesions have been noted in patients as long as 24 months after initial evaluation [7]. Overall prognosis of visual acuity shows a positive trend. Ampiginous chorioretinopathy causes progressive destruction of retina while sharing features of both APMPPE and serpiginous choroiditis [9]. Its chronic nature causes poor visual prognosis, and recurrences are common [4,9-14].

We describe three young Caucasian patients over an 8-month period, who presented with a distinct posterior uveitis similar to relentless placoid chorioretinitis but experienced an acute, non-recurrent clinical course similar to APMPPE. Four of five eyes involved had a final visual acuity of 20/40 or better, and 100% of the patients assayed had HLA-A3 and HLA-C7 association. Due to the unique clinical presentation, acute clinical course without recurrences, HLA association, and good visual prognosis, we believe they represent a new clinical entity called *panretinal acute multifocal placoid pigment epitheliopathy* or PAMPPE.

### Methods

We describe a cluster of young patients seen between April and December of 2006. All lived in central Ohio and were between the ages 16 and 27 (Table 1). The three patients were examined and followed with Snellen visual acuities, biomicroscopy, fundus examination, and photography for a period of at least 4 years. Fluorescein angiography and optical coherence tomography (OCT) were performed as needed. All patients were evaluated for known causes of posterior uveitis with uveitis review of systems, physical examination, and laboratory testing. The following laboratory testing evaluated in all three individuals included complete blood count, basic metabolic panel, liver function panel, erythrocyte sedimentation rate, C-reactive protein, blood cultures, HIV testing, Lyme titers, angiotensin converting enzyme, rapid plasma reagent, fluorescent treponemal antibody absorption test, tuberculin skin test (purified protein derivative), herpes simplex types I and II serology, anti-nuclear antibody, toxoplasma titers, and chest X-ray. One patient underwent brain MRI. Human leukocyte antigen (HLA) testing was performed in all three consenting patients. This is a retrospective review of their clinical cases. All patient information and clinical photographs were permitted by the patient with written and signed consent from each patient.

**Table 1:** PAMPPE

**Patient no./sex/race/age**	**Eye**	**Initial visual acuity**	**Medical history**	**Duration of activity (weeks)**	**Treatment**	**Final visual acuity**	**HLA typing results**
1/F/C/27	R	20/200	Ulcerative colitis, non-specific URI	6	None	20/20	A 2/3
	L	20/60				20/20	B 7/44
							C 5/7
2/M/C/17	R	20/400	HA, GI, fever, MRI	8	Prednisone, Subtenon's triamcinolone (Bristol-Myers Squibb Company, NY, USA)	20/200	A 1/3
							B 8/35
							C 4/7
3/M/C/16	R	20/400	Non-specific URI	4	Prednisone, PF 1%, oral ciprofloxacin	20/40	A 2/3
						20/20	B 7/57
	L	20/50					C 6/7

C, Caucasian; HA, headache; GI, gastrointestinal symptoms; MRI, magnetic resonance imaging; URI, upper respiratory tract infection; PF, Pred Forte (Allergan, Inc., CA, USA).

#### Case 1

A 27-year-old Caucasian woman presented with sudden decrease of vision in the left eye 3 weeks prior. Her medical history was remarkable for ulcerative colitis and non-specific upper respiratory tract infection approximately 6 weeks prior. A uveitis review of systems and laboratory evaluation were unremarkable. Initial visual acuities were 20/20 OD and 20/60 OS falling to 20/200 OD and 20/60 OS over several weeks. Intraocular pressures were 15 mmHg OD and 14 mmHg OS. Anterior segment findings were within normal limits OU, and no cell or flare was present.

**Figure 1:**
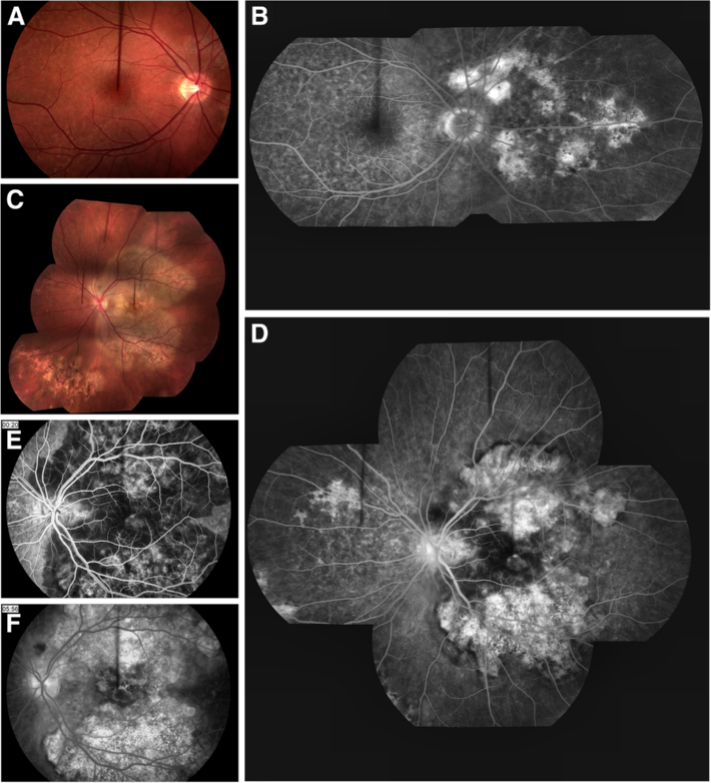
**Case 1.** A 27-year-old Caucasian female with a reduced initial VA of 20/200 OD and 20/60 OS due to PAMPPE. (**A**) Appearance of right fundus upon initial examination with active lesions present in the periphery. (**B**) Fluorescein angiogram montage demonstrating the atypical presentation and diffuseness of the lesions located in the posterior pole OD. (**C**) Fundus photograph montage of left eye taken upon initial evaluation showing areas of active lesions along with hyperpigmented areas of scarring in the RPE of the posterior pole. (**D**) Fluorescein angiogram montage demonstrating the atypical presentation of the lesions located in the posterior pole of the left eye. (**E**) Fluorescein angiogram of left eye at 20 s showing early phase/hypofluorescence or blockage upon initial evaluation along with (**F**) late phase/hyperfluorescence or staining of the active lesions at 5 min and 56 s.

Initial dilated fundus examination revealed multifocal, grayish/white lesions scattered throughout the fundus of both eyes (Figure 1). The left eye had a large lesion from arcade to arcade in the posterior pole along with lesions in the peripheral retina. Fundus photography and fluorescein angiography demonstrated ‘block early stain late’ appearance (Figure 1). The right eye developed new lesions over a 2-week period.

The patient was seen at 2, 4, 6, and 12 weeks, 6 months, and one year for re-evaluation. After 1 year, the patient was seen yearly for routine evaluation for 4 years total. The patient did not receive any therapy or treatments. Visual acuity improved to 20/20 OU by week 12 follow-up visit and remained 20/20 at her 12-month follow-up and subsequent evaluations. Fundus examination revealed widespread and scattered areas of depigmentation, and both fine and course clumping of the pigment epithelium located throughout the posterior pole and the periphery in both eyes without active disease were noted by 4 weeks. HLA testing was performed, and class I antigens HLA-A3 and HLA-C7 were identified (Table 1).

#### Case 2

**Figure 2:**
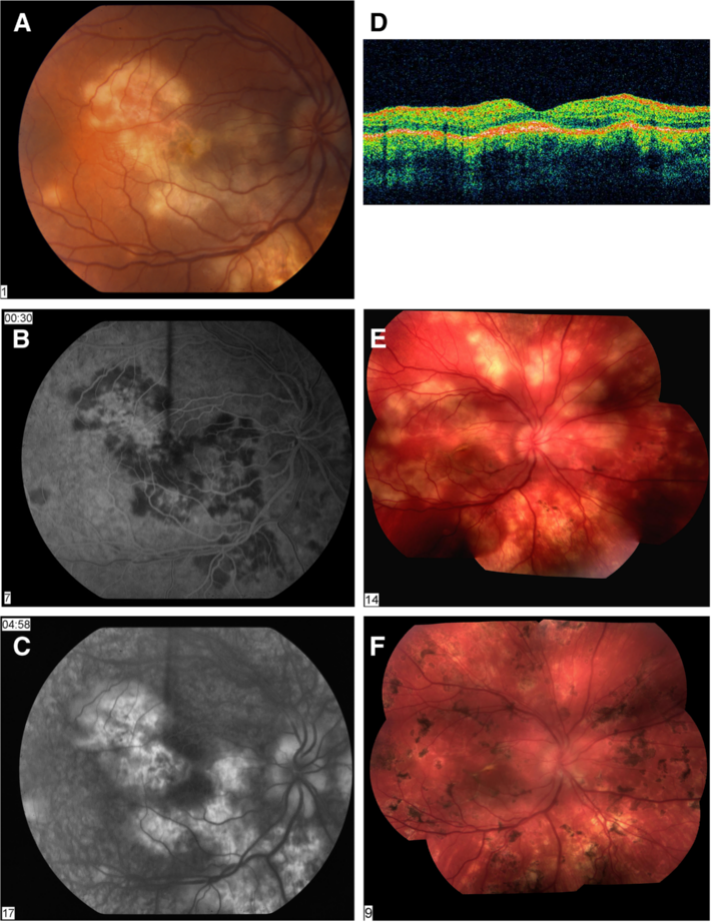
**Case 2.** A 17-year-old Caucasian male with visual acuity reduced to 20/400 OD at initial examination due to PAMPPE. (**A**) Fundus photography at initial evaluation with an atypical, panretinal, and diffuse presentation. (**B**) Fluorescein angiogram of right eye at 30 s showing the characteristic early phase/hypofluorescence or blockage upon initial evaluation along with (**C**) late phase/hyperfluorescence or staining of the active lesions at 4 min and 58 s. (**D**) OCT examination during the acute phase with thickening of the RPE. (**E**) Fundus photograph montage at the time of initial examination demonstrating the atypical, panretinal, and diffuse active lesions. (**F**) Follow-up examination after 8-week duration showed the presence of diffuse scarring in the RPE.

This is a 17-year-old Caucasian male with a 1.5-week history of decreased visual acuity, photophobia, and mild ocular pain in his right eye. He had previously been in the emergency room for a viral condition with GI pain, vomiting, and fever approximately 2 months prior to the onset of visual symptoms. Uveitis survey and past medical history were remarkable for mild headaches and psoriasis. Laboratory evaluation was unremarkable including normal MRI, except for an elevated CRP at 12.3. Best-corrected visual acuity was 20/400 OD and 20/20 OS. Intraocular pressures were 8 mmHg OD and 5 mmHg OS. Anterior segment examination found 1+ cells in the anterior chamber and 2+ cells in the vitreous cavity of the right eye; left eye, within normal limits. Dilated fundus examination found multiple, diffuse, creamy-white lesions in both the posterior pole and peripheral retina (Figure 2). Fundus photography and fluorescein angiography demonstrated a ‘blocking early, staining late’ pattern. Due to the intraocular inflammation, he was treated with oral prednisone.

The patient was followed-up at 2, 4, 6, 8, and 12 weeks, 6 months, and 1 year for re-evaluation. The patient was then followed yearly for a total of 4 years. The lesions evolved over an 8-week period, with increasing size and number of the retinal lesions, and he received a Subtenon's injection of triamcinolone 40 mg. Two weeks after the initial evaluation, the patient stated visual acuity had improved, with best-corrected visual acuity at follow-up to be 20/200 OD. The anterior segment had less inflammatory cell, and the patient was continued on oral prednisone for a 6-week period with slow taper. Following re-examination after 8 weeks, visual acuity and examination revealed further improvement. Fundus examination noted diffuse retinal pigment epithelial scarring throughout the retina (Figure 2). The patient has not experienced any recurrences.

OCT examination during the acute phase showed thickening at the RPE, along with normal foveal depression. HLA testing identified class I antigens HLA-A3 and HLA-C7.

#### Case 3

**Figure 3:**
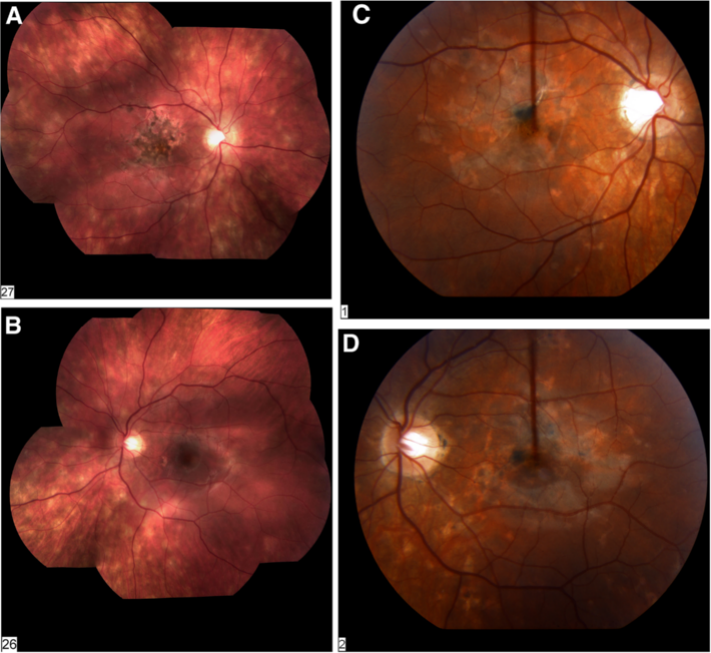
**Case 3.** A 16-year-old Caucasian male with visual acuity reduced to 20/400 OD and 20/50 OS at initial examination. (**A**) Montage fundus photograph of the right eye demonstrating a diffuse distribution of active lesions located extensively throughout the entire retina, along with scarring of the RPE in the area of the macula. (**B**) Montage fundus photograph of the left eye showed creamy white active lesions diffusely scattered. Panels (**C**) and (**D**) show the fundus photography OU after 5 months, showing no recurrences of active lesions and the presence of diffuse scarring.

This is a 16-year-old Caucasian male with a 2-week history of decreasing visual acuity. Past medical history was unremarkable, although he did report non-specific recent upper respiratory tract infection. Uveitis survey and laboratory evaluation were unremarkable. At time of ocular examination, visual acuity was 20/400 OD and 20/50 OS. Intraocular pressure was 15 mmHg OU. Dilated fundus examination of the right eye found multifocal placoid hypopigmented lesions at the RPE. Examination of the left fundus showed a smaller number of lesions (Figure 3). Fundus photography and fluorescein angiography demonstrated multifocal and diffuse lesions scattered throughout the posterior pole and peripheral retina of both eyes.

Treatment plan was 30 mg prednisone taper, and continuation of oral ciprofloxacin BID started previously by PCP for upper respiratory tract infection. Follow-up examinations took place at the interval of 2, 4, 6, 8, and 12 weeks, and 6 months, and 1-year duration. The patient was then followed yearly for a total of 4 years. The lesions resolved over a 4-week period with improved visual acuities to 20/60 OD and 20/20 OS by 12 weeks and 20/40 OD and 20/20 OS by 6 months (Figure 3). There have been no recurrences. HLA testing was performed, and HLA-A3 and HLA-C7 were present.

### Results

Our patients demonstrated a unique clinical presentation with acute retinal lesions similar to APMPPE but with more widespread presentation of multiple lesions in the peripheral retina. All patients were young, Caucasian, and exhibited an acute loss of vision, two of them bilaterally. All three patients reported prior illness. Ophthalmoscopy and fundus photography demonstrated a diffuse distribution of lesions located throughout the retina. On fluorescein angiography, the lesions showed a characteristic blocking-early-and-staining-late pattern similar to APMPPE. The average duration of activity was 6 weeks (range 4 to 8 weeks), and there were no recurrences. Four of five affected eyes had visual improvement of 20/40 or better. All were HLA-A3- and HLA-C7-positive. OCT during the acute stage in one patient found thickening in the RPE.

### Discussion

We present three patients whom we believe have a previously undescribed clinical entity called panretinal acute multifocal placoid pigment epitheliopathy or PAMPPE. The patients presented over an 8-month period in central Ohio. All were young, Caucasian, and previously healthy except one patient with inflammatory bowel disease. All patients had preceding viral-like prodrome. There were more males (2:1), the disease was bilateral in two of the three patients, and the clinical course was acute lasting 4 to 8 weeks. All three patients presented with sudden loss of vision. Ocular examination revealed multi-focal, creamy-white lesions at the level of the RPE distributed both in the posterior pole and peripheral retina. Fluorescein angiography demonstrated block-early-and-stain-late pattern. The disease was not progressive or recurrent. All patients underwent HLA testing, and 100% of the tested subjects were positive with HLA-A3 and HLA-C7. Two subjects (66%) were positive with HLA-A2 and HLA-B7. The visual prognosis was good with improvement in all subjects with 20/40 or better vision in four of the five involved eyes.

APMPPE generally affects young individuals between the age of 20 to 50 years, without gender predisposition [5]. The etiology of APMPPE is still unknown. Gass postulated that due to the pattern and rapidity of both, the development and resolution suggest that it represents an acute pigment epithelial response to local infectious agents [15]. The lesions in APMPPE are classically confined to the posterior pole and have been associated with HLA-B7 [5]. Our patients demonstrated retinal lesions similar to those in APMPPE but had more widespread and panretinal distribution. Patients with RPC have more lesions than those with APMPPE and are persistent with prolonged periods of activity along with relapses being common, resembling the clinical course similar to serpiginous choroiditis [7]. The clinical course of patients with RPC ranges from 5 to 24 months. Ampiginous chorioretinopathy is a cross between APMPPE and serpiginous choroiditis, and presents with RPE hyperpigmentation and atrophy in inactive healing lesions [9]. Previous reports have also associated serpiginous with HLA-B7 [5]. Similar to RPC, ampiginous choroidopathy is chronic or recurrent [9]. Visual prognosis is poor in ampiginous choroidopathy. In our three young, Caucasian patients, the mean duration of active lesions was 6 weeks, not recurrent, and the visual prognosis was good.

We found that HLA-A3 and HLA-C7 were common in all three (100%) of the patients. The estimated gene frequency in the Caucasian population for HLA-A3 and HLA-C7 has been reported to be 13% and 16%, respectively. APMPPE and serpiginous have been reported to have 40% association with HLA-B7. HLA-B7 has a gene frequency of 12% in the Caucasian population. Two of the three patients with PAMPPE (66%) had HLA-B7 and HLA-A2.

These major histocompatibility (MHC) associations and similarities found in our patients suggest a common susceptibility and pathophysiologic mechanism for PAMPPE. A postulated theory for HLA-disease association would suggest these common MHC antigens (HLA-A3, A2, B7, and C7) predispose these individuals when combined with prodromal virus or viral particles. The resulting cell surface complex would then be recognized as foreign by the host's immune complex. This could then result in an autoimmune response, directed at tissues bearing these modified antigenic moieties. One of our patients had an OCT during the acute phase showing a thickening of the RPE, suggesting an immune response.

The most appropriate method of treatment for PAMPPE is still to be determined. One of our patients received no treatment. Two out of the three patients were treated with oral prednisone, and one patient received Famvir (Novartis AG, Basel, Switzerland) and Pred Forte. The overall prognosis showed a positive trend in all patients. Only one patient experienced minimal improvement. Four of the five affected eyes had visual improvement of 20/40 or better. Three of the five eyes examined had a final visual acuity of 20/20.

We present a retrospective series of patients experiencing an atypical clinical entity resembling relentless placoid chorioretinitis but with a shorter duration of activity similar to APMPPE, no recurrences, good visual prognosis, and HLA-A3/C7 association affecting Caucasian young adults. All three patients experienced preceding viral illness, with no other environmental commonalities. To our knowledge, no previous literature has expressed any similar discoveries. We believe this to be a new clinical entity, which we are calling *panretinal acute multifocal placoid pigment epitheliopathy* or PAMPPE.

## Competing interest

The authors' declare that they have no competing interests.

## Authors' contributions

KRB and EMO drafted the manuscript, participated in the conception, coordination and design of the study, and performed the statistical analysis. Both authors read and approved the final manuscript.
